# Systematic review of health literacy champions: who, what and how?

**DOI:** 10.1093/heapro/daad074

**Published:** 2023-07-20

**Authors:** Julie Ayre, Michael Zhang, Dana Mouwad, Dipti Zachariah, Kirsten J McCaffery, Danielle M Muscat

**Affiliations:** The University of Sydney, Faculty of Medicine and Health, Sydney School of Public Health, Sydney Health Literacy Lab, Sydney, NSW, Australia; The University of Sydney, Faculty of Medicine and Health, Sydney School of Public Health, Sydney Health Literacy Lab, Sydney, NSW, Australia; Health Literacy Hub, Western Sydney Local Health District, Sydney, NSW, Australia; Multicultural Health, Western Sydney Local Health District, Sydney, NSW, Australia; The University of Sydney, Faculty of Medicine and Health, Sydney School of Public Health, Sydney Health Literacy Lab, Sydney, NSW, Australia; The University of Sydney, Faculty of Medicine and Health, Sydney School of Public Health, Sydney Health Literacy Lab, Sydney, NSW, Australia

**Keywords:** champion, health literacy, organizational health literacy, implementation

## Abstract

Health literacy is an important aspect of equitable, safe, and high-quality care. For organizations implementing health literacy initiatives, using ‘change champions’ appears to be a promising strategy. This systematic review aimed to identify the empirical and conceptual research that exists about health literacy champions. We conducted the systematic literature search using MEDLINE, Embase, CINAHL, Scopus, and PubMed, with additional studies identified by searching references and citations of included studies and reviews of organizational health literacy. Seventeen articles were included in the final review (case studies, *n* = 9; qualitative research, *n* = 4; quasi-experimental, *n* = 2; opinion articles without case studies, *n* = 2). Using JBI critical appraisal tools, most articles had a high risk of bias. Often champions were not the focus of the article. Champions included staff across frontline, management, and executive levels. Only five studies described training for champions. Key champion activities related to either (i) increasing organizational awareness and commitment to health literacy, or (ii) influencing organizational strategic and operational planning. The most common output was ensuring that the organization’s health information materials met health literacy guidelines. Articles recommended engaging multiple champions at varying levels within the organization, including the executive level. Limited funding and resources were key barriers. Two of four articles reported positive impacts of champions on implementation of health literacy initiatives. Overall, few of the articles described health literacy champions in adequate detail. More comprehensive reporting on this implementation strategy and further experimental and process evaluation research are needed to progress this area of research. This systematic review was registered with PROSPERO (CRD42022348816).

**Contribution to Health Promotion**

- Health literacy is important for developing safe and accessible health promotion initiatives and resources. However, uptake of health literacy practices within organizations is often poor.
- Champions may be a useful strategy for improving uptake of health literacy practices within in a health organization. This review identified 17 articles about health literacy champions, most with high risk of bias.
- Champion activities focused on: (i) raising awareness and commitment to health literacy; or (ii) changing organizational strategies and processes. Organizations may benefit from having health literacy champions at different levels within the organization, including the executive level. However, more research is needed.

## INTRODUCTION

Health literacy is an important consideration for any health organization that seeks to provide equitable, safe, and high-quality care. This is clearly demonstrated across a range of health outcomes: low health literacy is associated with higher mortality, morbidity, medication errors, and rates of hospitalization and emergency department visits (Berkman *et al.*, 2011). Though these associations relate to an individual’s health literacy (i.e. skills to access, understand, appraise, and use health information and services), we must recognize the critical role that health organizations also play (Nutbeam and Muscat, 2021). For example, organizational structures and resources affect how easily people can navigate a health service, the quality of health information provided to patients, and extent that staff are trained in health literacy concepts and communication skills (Farmanova *et al.*, 2018).

For organizations implementing health literacy initiatives, using ‘change champions’ appears to be a promising strategy. The Consolidated Framework for Implementation Research (CFIR) defines champions as ‘*individuals who dedicate themselves to supporting, marketing, and* “*driving through an [implementation]*”*, overcoming indifference or resistance that the intervention may provoke in an organization*’ (Greenhalgh *et al.*, 2004; CFIR Research Team-Center for Clinical Management Research, 2022). A recent scoping review identified change champions as one of four critical factors for implementing organizational health literacy interventions (Kaper *et al.*, 2021). Similarly, a 2018 systematic review on the same topic identified the absence of a change champion as one of 13 key barriers (Farmanova *et al.*, 2018). These findings reflect broader healthcare implementation research. For example, reviews show ‘generally positive’ evidence that champions contribute meaningfully to implementation efforts, and implementation science experts consider ‘identifying and preparing champions’ an important and highly feasible implementation strategy that should be prioritized (Waltz *et al.*, 2015; Miech *et al.*, 2018; Lennox *et al.*, 2020).

However, there is surprisingly little research defining the concept of ‘change champion’, and evaluating the impact of change champions on healthcare implementation efforts. Often research on champions is only descriptive in nature, lacking in detail, or the findings are embedded within broader, complex implementation efforts that cannot isolate the individual effect of the champions (Miech *et al.*, 2018; Shea, 2021; Santos *et al.*, 2022). To illustrate, two reviews on champions in healthcare implementation reported that the vast majority of articles only considered champions in terms of presence or absence [more than 90% of 199 articles (integrative review; Miech *et al.* (Miech *et al.*, 2018)], 71% of 35 articles [systematic review of quantitative research only; Santos et al. (Santos *et al.*, 2022)]. Santos and colleagues’ (2022) systematic review of quantitative research related to healthcare champions reported that though champions were related to increased use of healthcare innovations at an organizational level (i.e. policies and processes), there was inconsistent evidence about whether champions were also related to improvements in provider’s attitudes and knowledge, use of innovations, and patient outcomes.

This lack of detailed research on champions is also observed in systematic reviews of organizational health literacy, all of which highlight the role of champions, but bear little detail about how to implement this strategy effectively. For example, there was no detail about who champions were, how champions were identified, what training they received, and what activities they engaged in as champions (Farmanova *et al.*, 2018; Lloyd *et al.*, 2018; Kaper *et al.*, 2021). Notably, none of these reviews have assessed the quality of available evidence, rendering it difficult to understand the state of the science in this emerging field and how it can best be progressed.

It is also possible that these reviews of organizational health literacy overlooked some articles relating to health literacy champions given the search terms they used. This oversight is important because the context of *health literacy* may be different to that of other healthcare champions. For example, health literacy initiatives can vary greatly in scale (e.g. within a specific department vs. initiatives that span across multiple services and sites), and often involve partnership across disciplines, professions, sectors, and community organizations (Sørensen *et al.*, 2021).

To capture the state of the literature and identify evidence to inform practice relating to health literacy champions, we undertook a systematic review to identify the empirical and conceptual research that exists about health literacy champions, including descriptive accounts (e.g. of their roles, responsibilities, selection, and training), evaluations of training and implementation, and relevant models and theoretical frameworks (Munn *et al.*, 2018). Although we can think about health literacy champions as including people who operate across sectors or services, and individuals who are exemplars of health literate practice, in this study we focus on health literacy champions who seek to improve the health literacy practices of other staff members within their organization.

## METHODS

### Protocol and registration

This systematic review was registered with the international Prospective Register of Systematic Reviews (PROSPERO) (CRD42022348816). No amendments to the registered protocol were required, except that case study, and text and opinion articles were deemed high risk of bias in line with Burns *et al.* (Burns *et al.*, 2011) (see in section 2.7). The review is reported in accordance with the Preferred Reporting Items for Systematic reviews and Meta-analyses 2020 statement (Page *et al.*, 2021). This study was based exclusively on published literature. As such, no ethics approval was required.

### Review question

What empirical and conceptual research exists about health literacy champions, including descriptive accounts, evaluations of champion effectiveness, evaluations of champion training and implementation, and relevant models and theoretical frameworks?

### Inclusion and exclusion criteria

For this review, we included English-language articles published in peer-reviewed journals or published books that examined the concept of a health literacy champion. In line with the CFIR definition (CFIR Research Team-Center for Clinical Management Research, 2022), health literacy champions was taken to refer to staff within an organization who are involved in implementation, delivery, or provision of a health literacy initiative that seeks to improve the health literacy practices in other staff members. No limits were set for date of publication.

Studies were excluded if they met any of the following criteria:

1. Mentioned the concept of health literacy champion as a future direction only and text about champions was not directly related to aims, methods or results of the manuscript.
2. Concerned with mental health literacy champions only.
3. Involved patient/community/peer-led education initiatives.
4. Focused on health literacy improvement in patient or community populations, rather than improvement in health literacy practices in an organization.

### Search strategy

A database search of MEDLINE, Embase, CINAHL, Scopus, and PubMed was conducted on 8 August 2022. Article titles, abstract, and keywords were searched using the following search string, based on other reviews of healthcare champions [see e.g. (Miech *et al.*, 2018; Wood *et al.*, 2020)]:

(‘health literacy’ or ‘health literate’) AND (‘champion*’ or ‘change agent*’ or ‘opinion leader*’ or ‘liaison*’ or ‘liason*’ or ‘ambassador*’ or ‘implementation leader*’ or ‘emergent leader*’ or ‘promoter*’ or ‘advocate*’).

Where possible MeSH search terms were used (see Supplementary Appendix).

Conference abstracts that appeared during the database searching were excluded but potentially relevant full-text articles relating to these conference abstracts were identified and screened. Systematic reviews on organizational health literacy were also identified and examined for any potentially relevant articles. Additionally, a snowballing approach was used which involved searching the reference lists and citations (‘cited by’ in Google Scholar) of eligible articles.

### Study selection process

After duplicates were removed, titles and abstracts were independently screened by two authors (JA and MZ) for full-text screening. All full texts were also independently screened for inclusion by these authors (JA and MZ). Any disagreements during this process were resolved through discussion between study authors.

### Data extraction and synthesis

Data for each article that met the inclusion criteria were independently extracted by two authors (JA and MZ). Extracted data was compared by the two authors and any differences were resolved through discussion with KM. Data extracted included year of publication, aims, study setting and design, interventions implemented, details about the health literacy champions (role, responsibilities, selection, training and effectiveness, and any potential facilitators or barriers to successful championship). Following data extraction, patterns across the data were explored and synthesized in narrative form (Popay *et al.*, 2006). Given the lack of quantitative data and limited detail, even in qualitative research, we did not seek to undertake subgroup analyses or sensitivity analyses. Findings about effectiveness were only synthesized for studies with low risk of bias (Boutron *et al.*, 2019).

### Quality appraisal

All full texts included in the data extraction process were assessed for risk of bias by two authors (JA and MZ) using standardized critical appraisal tools from JBI (https://jbi.global/critical-appraisal-tools). Depending on the study design, different JBI critical appraisal tools were used. These included the Checklist for Qualitative Research, and the Checklist for Quasi-Experimental Studies (see Supplementary Appendix). All Text and Opinion articles were considered high risk of bias. For case study designs, as there was no JBI critical appraisal tool for these study types, these studies were assessed as high risk of bias. These categorisations for text and opinion and case study texts are in line with well-established levels of evidence related to risk of bias (Burns *et al.*, 2011).

When articles contained multiple study design components, a checklist was utilized for all study designs relevant to health literacy champions. Using these tools, studies were categorized as: low risk of bias if most criteria were fulfilled and done well, moderate risk of bias if some of the criteria were fulfilled, or high risk of bias if most criteria were not done or done poorly. Discrepancies in ratings between the two authors were resolved through discussion.

## RESULTS

### Study details

**Fig. 1:**
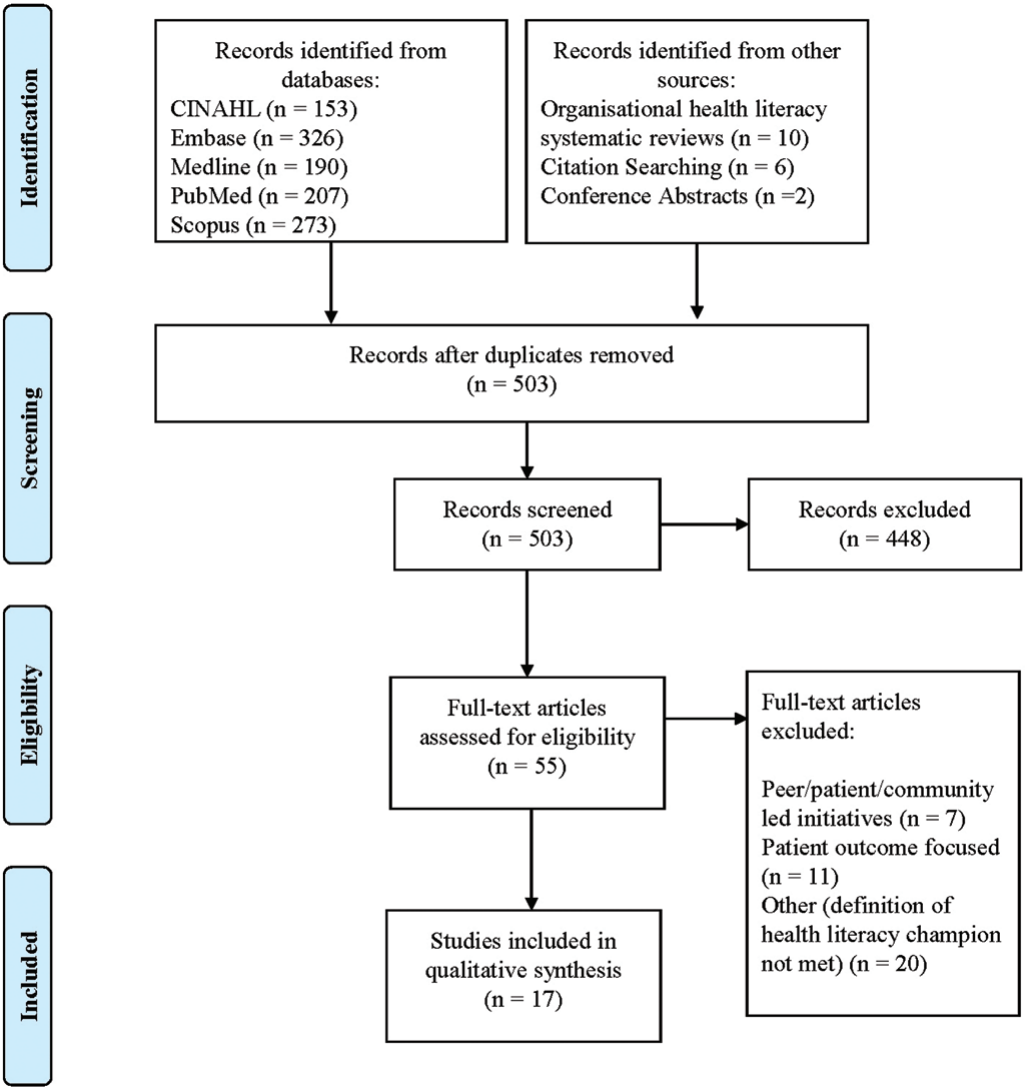
Preferred reporting items for systematic reviews and meta-analyses (PRISMA) flow diagram of the study selection process. Adapted from Moher *et al.* (Moher *et al*., 2009).

We retrieved 1149 articles from the database searches, and 18 from additional search methods (Figure 1). After removal of duplicates and screening by title and abstract, 55 full-text articles were screened for full-text inclusion. Articles were excluded if champions were community members rather than staff (*n* = 7), if they reported on health literacy improvement in patient or community populations, rather than improvement in health literacy practices in an organization (*n* = 11), or if the review’s definition of champion was otherwise not met, that is, the champion did not influence others within their organization (*n* = 20). Seventeen articles met our inclusion criteria and were included in the final synthesis.

### Study characteristics and risk of bias assessment

The 17 studies identified in the review are described in Table 1. With two exceptions, health literacy champions were not the primary focus of the included research articles and were mentioned as one aspect of implementation of a health literacy intervention or initiative. Only two articles focused primarily on health literacy champions (Brach *et al.*, 2014; Sørensen, 2021). These were both of the ‘Text and Opinion’ article type, with one providing additional case studies (Sørensen, 2021). Overall, nine articles adopted a case study design and provided an account of how organizational health literacy was introduced in an organization, with health literacy champions playing some part in this process (all high risk of bias). Four articles reported qualitative research investigating how health literacy practices (Adsul *et al.*, 2017; Howe *et al.*, 2020) or tools (Mabachi *et al.*, 2016; Kaper *et al.*, 2019) had been implemented within an organization (three low and one moderate risk of bias). Two studies used quasi-experimental designs to evaluate the implementation of a health literacy intervention in clinical settings (O’Neal *et al.*, 2013; Morrison *et al.*, 2021), although effects of champions were not isolated from the broader intervention (one low, one high risk of bias). Five articles were categorized as ‘Text and Opinion’ (two without accompanying case studies) and primarily provided a conceptual account of how organizations can improve their health literacy practices.

**Table 1:** Characteristics of included studies

First author (year)	Setting	Study aims	Intervention	Who were the HL champions?	HL champion role, responsibilities, activities performed	Risk of bias
Qualitative studies						
Adsul (2017)	US low-resourced health care organizations	Identify how health care organizations serving underserved populations adopt and implement health literacy related changes	Health literacy quality improvement program	Staff who were familiar with all aspects of the organization, recognized need for improvements in patient care.	Lead implementation/ facilitate change of health-literacy practices in organization (‘program champion’)	Moderate
Howe (2020)	US (Texas) hospitals	Describe key organizational leaders’ and clinicians’ perspectives on how health care systems were adopting health literate policies and practices that address the 10 attributes of a health literate healthcare organization	None described	Out of 13 services, only 1 had an appointed dedicated health literacy position; 1 additional example of a clinical nurse (frontline worker) who was an emergent champion	• Involve patients in information processes e.g. development and review of booklets • Delivering patient education in a creative manner e.g. using smartphones and tablets	Low
Kaper (2019)	Ireland, Netherlands	Assess the implementation fidelity, moderators (barriers and facilitators), and long-term impact of organizational HL interventions in hospitals	Organizational HL intervention, including HL audit, implementation coordinators, project committees	Appointed staff including senior nurses, policy advisor, communications officer, communication consultants	• Coordinate implementation process • Embed health literacy in working procedures and professional practice	Low
Mabachi (2016)	US primary care practices	1.Examine utility of the health literacy universal precautions toolkit for primary care practices 2. Identify possible refinements that would enhance the value of the Toolkit as a resource for primary care practices.	Universal precautions toolkit with support from contracted external health literacy experts	1. Appointed health literacy team leaders 2. Practice leaders (e.g. management level)	• Team leaders: oversee implementation • Practice leaders: directly involved in planning and implementation, publicly empower the health literacy team to carry out project activities	Low
Quasi-experimental						
Morrison (2021)	US emergency departments	Improve the distribution, satisfaction, and understanding of asthma education and discharge instructions in parents of children with asthma exacerbation in the ED	Process maps to understand workflows and inform the health literacy initiative (4 stages: education, integration, refresher and sustainment)	Physician and education expert, 4 ED nurse champions and a quality improvement specialist.	• Develop an implementation strategy and new materials • Trial new materials/strategy • Work closely with IT and quality improvement staff • Peer to peer education (nurses) about educational materials and health literacy-focused communication	Low
O’Neal (2013)	US community pharmacies	1. Pilot, evaluate and adapt Pharmacy Health Literacy Assessment Tool 2. Describe use of health literacy practices from patient, staff, and independent auditor perspectives 3. Evaluate the effect of a low-intensity educational health literacy awareness program 4. Identify opportunities to improve health literacy sensitive practices in the community pharmacy environment.	AHRQ pharmacy HL assessment tool; 2-hour HL literacy training workshop for pharmacists delivered by nationally recognized expert	Pharmacy staff who attended the training	Initiate literacy sensitive practices at their pharmacy (the extent of this was up to the champion)	High
Case studies						
Allott (2018)	Health and community services in Australia	Describe how a ‘system approach’ to health literacy responsiveness created change in regional health and community services	1. Health literacy training course (8 months) 2. Executive level workshop 3. Community of practice (meetings and networks) 4. Alliance to facilitate peer support, problem-solving, and regional action	Not specific if emergent or appointed. Some champions had attended the training course	Champion health literacy principles and practices within the organization	High
Brach (2017)	US health care organizations that have pioneered a systematic approach to health literacy	Explore the process for organizations to become health literate health care organizations	None described	HL champions can be: 1. Organizational leaders (e.g. CEO’s) 2. Formal health literacy liaisons (appointed) 3. Staff who have informally become standard bearers for health literacy in the course of their jobs (emergent)	Organizational leader: • Initiate research • Engage other organizational leaders • Establish a HL taskforce • Implement policies in hospitals Formal HL liaison: • Ensure health information is easy to access, understand, and use • Ensure system-wide processes take health literacy into account • Promote system-wide health literacy standards • Develop a system-wide strategic plan for health literacy Emergent: • Advocate through passion for improving patient education and written materials • Raise health literacy to the top levels of an organization	High
Briglia (2015)	US federally qualified health center	Provide steps for becoming a health literate organization	None described	Contracted health literacy program manager	• Lead HL initiative • Create and communicate a change vision, generate staff awareness about HL • Establish a HL task force • Implement staff training and agency-wide HL policies • Ensure all patient materials meet HL guidelines	High
Erikson (2019)	US health services (state level)	Outline how an adult basic education coalition developed a state-wide health literacy coalition	None described	Emergent champion was an influential physician who joined the board of directors for Wisconsin Literacy Inc	• Deliver presentations on HL at state and national conferences • Create opportunities and influence conversations in health care settings	High
Finlay (2019)	Acute and community health services in a regional area of Australia	Report on outcomes of a project (Gippsland Health Literacy Project) designed to educate local health services staff about health literacy and provide tools and techniques for health literacy implementation in service delivery	1. An introductory health literacy forum 2. A 2-day health literacy short course 3. Training in the use of specific health literacy tools 4. Completion by participants of individual ‘plan do study act’	Not described	Not described	High
O’Neill (2019)	US health services	Provide a case study/example model of health literacy change in a health system	None described	Emergent: 1. Nurse-educator from Nursing and Professional Development & Education department 2. Chief learning Officer Identified: 3. Medical Director of Patient and Family Health Education	Nurse: • Campaign for the issue of health literacy in leadership venues, for example, nursing councils and service line leader meetings • Organize Health Literacy Awareness Month events • Campaign to department director and Chief Learning Officer (member of CEO roundtable) • Assess HL practices, present findings to senior leadership Chief learning officer: • Develop system-wide organizational processes to create uniform messaging about health literacy • Consolidate education content vendors across the organization • Allocate more personnel resource to support these changes and future efforts • Establish MD as additional co-advocate to increase credibility, perspective and relatability to conversations about health literacy practices Medical director: • Form a governance group including consumers	High
Shoemaker (2013)	US pharmacies	Identify facilitators and barriers to uptake and implementation of AHRQ’s health literacy tools	AHRQ pharmacy health literacy tools	1. Part-time pharmacy staff (e.g. clinical faculty, students) 2. Pharmacy staff with designated responsibilities besides dispensing (e.g. patient care) 3. Full-time Director of Pharmacy Operations	Implement the tool	High
Sørensen (2021)	Health organizations (international focus)	Explore health literacy championship and how health literacy champions are characterized and nurtured as change-agents for the development of health literate organizations, settings and societies	Gives example of Health Literacy Champion Process (for Nebraska LHD), in which an organization can apply to be a designated Health Literacy Champion to incentivise implementation of HL practices	Describes champions as leaders and/or decision-makers in the organization who have the influence needed to approve or put the plan into action. Distinguishes this role from allies (supporters), and workgroup members (day to day HL activities) (CDC case study). Champions may be internal or external to the organization Characteristics include patience, endurance, and long-term vision (‘enthusiastically and relentlessly defends and fights for the cause of health literacy to the benefit of people and societies at large’)	•‘Induce’ and develop the change in organizational thinking •Explain the necessity to perform a change of practice •Generate general or public interest to effect change	High
Vellar (2017)	Australian regional health service	Describe how health service embedded health literacy principles over a 3-year period	Three phases to implementation of a HL Framework: 1. Literature review and critical reflection on critical incidences 2. Organizational commitment and consultation process 3. Piloting health literacy strategies for system-wide improvements, including HL ambassador (HLA) program that trains staff to lead their teams on how to partner with consumers to develop plain English resources	1. Appointed health literacy ambassador 2. Senior executive leadership	HL ambassador: lead development of plain English resources for patients Senior executive leadership: advocate for HL within their level of influence and advocate for HL as a quality and safety issue	High
Text and opinion						
Brach (2014)	US health services	Describe physician’s role in a creating a health literate organization	None described	Physicians within a health organization	• Promote integration of health literacy into strategic and operational planning, evaluation and quality improvement • Insist on HL training for all staff, and system redesign to meet needs of patients at all HL levels • Install mechanisms to obtain input from patients to improve services • Direct resources to where misunderstandings would have the most severe consequences • Promote price transparency to make out of pocket costs easy to understand and timely	High
Erlen (2004)	USA	Provide an overview of functional health illiteracy, identify related ethical concerns, and discuss selected, relevant nursing implications	None described	Nurses	• Increase awareness of health literacy amongst other health professionals • Develop protocol to assess individual health literacy • Direct healthcare staff to health literacy research articles and HL measures • Advocate for health literate patient education materials • Facilitate decision making: explain difficult information and translate technical language into language that patients understand (though this is pitched as working directly with patients)	High

### Outcomes

#### Who were the health literacy champions?

Health literacy champions were most often described in terms of their professional role. For example, champions included nurses (Erlen, 2004; Kaper *et al.*, 2019; O’Neill, 2019; Morrison *et al.*, 2021), physicians (Brach *et al.*, 2014; Erikson *et al.*, 2019; Morrison *et al.*, 2021), pharmacists (O’Neal *et al.*, 2013; Shoemaker *et al.*, 2013), medical residents (Shoemaker *et al.*, 2013), and staff involved in policy, communication and quality improvement (Kaper *et al.*, 2019; Morrison *et al.*, 2021). Another important group were champions in positions of leadership, including at the executive level (Shoemaker *et al.*, 2013; Mabachi *et al.*, 2016; Brach, 2017; Sørensen, 2021). Two studies described champions who were consultants or externally contracted staff with expertise in health literacy (Briglia *et al.*, 2015; Kaper *et al.*, 2019). Champions were also variously described ‘emergent’ (Erikson *et al.*, 2019; Sørensen, 2021) (i.e. staff who take on a champion role of their own accord due to their high commitment to the cause), or as staff ‘appointed’ to a champion role (Briglia *et al.*, 2015; Mabachi *et al.*, 2016; Kaper *et al.*, 2019). Sometimes emergent champions worked in services that did not initially value or engage in health literacy practices, for example, (Erikson *et al.*, 2019; O’Neill, 2019). However, this was not always the case. For example, Brach (Brach, 2017) discussed that CEO-level staff often became health literacy champions in part due to alignment with the organization’s mission and goals.

If more than one champion was present, a combination of both emergent and appointed champions were often involved (Brach, 2017; Shoemaker *et al.*, 2013; Vellar *et al.*, 2017; O’Neill, 2019; Howe *et al.*, 2020). Typically champions in a senior leadership position were ‘emergent’, whereas staff on the ground were either emergent or appointed (Shoemaker *et al.*, 2013; Mabachi *et al.*, 2016; Brach, 2017; Vellar *et al.*, 2017; O’Neill, 2019).

#### Champion training

Five studies described health literacy training programs for champions, which ranged in duration from a single 2-hr workshop (O’Neal *et al.*, 2013; Vellar *et al.*, 2017) through to 8 months of ongoing training (Allott *et al.*, 2018). Two studies described a continuation of learning through ongoing mentoring and collaborative support from other champions (Vellar *et al.*, 2017; Allott *et al.*, 2018). Of the five studies that included training, only two mentioned specific training in implementation skills in addition to general health literacy knowledge and skills (Finlay *et al.*, 2019; Morrison *et al.*, 2021). Kaper et al. (Kaper *et al.*, 2019) described how implementation skills were supplemented by ‘implementation coordinators’.

More than half of the articles (*n* = 9) did not describe any form of training for health literacy champions, with most of these focusing on emergent champions.

#### Health literacy champion activities, roles and responsibilities

Key activities (or roles and responsibilities) fell broadly into three categories: increasing health literacy awareness and organizational commitment; changing strategic and operational planning; and influencing frontline health literacy practices.

Generating awareness about health literacy was typically focused within the organization but occasionally extended beyond (Erlen, 2004; Briglia *et al.*, 2015; Erikson *et al.*, 2019; O’Neill, 2019; Sørensen, 2021). This encompassed communicating the change vision and advocating health literacy to organizational leaders. Three articles described that health literacy champions could seek to influence other organizational leaders to support health literacy initiatives or become health literacy champions themselves (Brach, 2017; Erikson *et al.*, 2019; O’Neill, 2019).

A second key activity was influencing strategic and operational planning (Brach *et al.*, 2014; Briglia *et al.*, 2015; Mabachi *et al.*, 2016; Brach, 2017; Kaper *et al.*, 2019; O’Neill, 2019). On a broad level this was described as changes to organizational policy (Briglia *et al.*, 2015; Brach, 2017) and processes (Brach, 2017; Kaper *et al.*, 2019). For example, for three studies this explicitly involved linking health literacy to existing quality improvement processes and IT services (Brach *et al.*, 2014; Vellar *et al.*, 2017; Morrison *et al.*, 2021).

Several articles highlighted the role of champions in influencing frontline health literacy practices. Six articles described the champion as ensuring that the organization’s health information materials met health literacy guidelines (Erlen, 2004; Briglia *et al.*, 2015; Brach, 2017; Vellar *et al.*, 2017; Howe *et al.*, 2020; Morrison et al., 2021). Three studies described how champions implemented health literacy training for staff (Brach *et al.*, 2014; Briglia *et al.*, 2015; Morrison *et al.*, 2021); three described establishing a health literacy task force, working group, or committee (Briglia *et al.*, 2015; Brach, 2017; O’Neill, 2019) (though with little detail about the aims of these groups); and three described advocacy and implementation of mechanisms to increase consumer engagement in the organization’s practices (Brach *et al.*, 2014; O’Neill, 2019; Howe *et al.*, 2020). Other activities included assessment of organizational health literacy practices (O’Neal *et al.*, 2013; Shoemaker *et al.*, 2013; O’Neill, 2019), or of individual (patient) health literacy (Erlen, 2004).

Two studies did not provide specific details and simply alluded to the champions leading implementation and advocating for health literacy (Adsul *et al.*, 2017; Allott *et al.*, 2018).

#### Potential facilitators and barriers to successful championship

Several studies identified the importance of support and commitment to health literacy initiatives from executive leadership (Mabachi *et al.*, 2016; Brach, 2017; Allott *et al.*, 2018; Finlay *et al.*, 2019; Howe *et al.*, 2020; Sørensen, 2021). Many also emphasized that health literacy champions cannot act in isolation, and recommended multiple champions at varying levels within the organization (Brach, 2017; Vellar *et al.*, 2017; Howe *et al.*, 2020; Sørensen, 2021). Further, champions can be supported by other groups within the organization; Sørensen (Sørensen, 2021) describes the CDC case study which depicts champions as working in unison with allies (who provide support/vision), and workgroup members (day to day planning and coordination).

Some studies described the importance of organizational awareness and commitment to health literacy *before* appointing health literacy champions (Mabachi *et al.*, 2016), for supportive policies and infrastructure to be in place (Brach *et al.*, 2014), and for a culture that fosters innovation and quality improvement (Sørensen, 2021).

Lastly, limited resources, lack of dedicated personnel and limited funding were often identified as barriers to effective health literacy champions (Shoemaker *et al.*, 2013; Brach, 2017; Howe *et al.*, 2020).

For appointed champions, bolstering commitment to health literacy may also be important. The authors of two studies proposed several examples of strategies that could strengthen this commitment: personal invitation to champion health literacy from a trusted source, that is, academic institution; awards and other incentives; and aligning champion activities with other goals (such as meeting residency requirements) (Shoemaker *et al.*, 2013; Sørensen, 2021). Shoemaker *et al*. (Shoemaker *et al.*, 2013) also suggested that providing ongoing support from health literacy experts helped strengthen the commitment of health literacy champions.

#### Effectiveness

Overall, four articles with low risk of bias reported on the effectiveness of champions. This included three qualitative studies and one quasi-experimental study. Two reported positive effects (Howe *et al.*, 2020; Morrison *et al.*, 2021). The remaining two articles reported neutral effects of health literacy champions (e.g. the champion was only one component of the health literacy initiative and was not identified as a critical factor) (Mabachi *et al.*, 2016; Kaper *et al.*, 2019). Both studies reporting positive effects involved emergent champions, and the third study did not report this characteristic; by comparison, the two studies reporting neutral effects involved appointed champions.

The quasi-experimental study explored a health literacy initiative to improve asthma education in a US emergency department (Morrison *et al.*, 2021). Champions were only one component of this initiative, and their unique effects were not reported. Study authors reported an increase in families receiving asthma education over a 12 month period for written (28–52%) and video materials (0–32%), although no statistical analysis was performed. The intervention did not result in changes to emergency department length of stay, length of discharge, or 30 day revisit rates.

## DISCUSSION

We identified 17 articles related to health literacy champions that were generally of high risk of bias. These articles provided only very limited detail about champions, in part because the articles focused on multi-component implementation efforts. Champions included staff on the ground (e.g. nurses, physicians, pharmacists), in administrative or management roles (e.g. quality improvement, senior nurses, communication), and in executive leadership roles. Few studies described training for health literacy champions, and those that did provided little detail. Key champion activities related to increasing organizational awareness and commitment to health literacy, influencing strategic and operational planning, and influencing frontline health literacy practices. The most frequently described influence on frontline practices was to ensure that the organization’s health information met health literacy guidelines. Articles recommended having multiple champions at varying levels within the organization, including the executive level. Limited funding and resources were identified as key barriers for health literacy champions. Two of four studies with low risk of bias reported that emergent champions may enhance implementation of health literacy initiatives. Further work is needed to isolate the effect of champions from other implementation strategies.

These findings highlight a clear lack of a foundational, rigorous evidence base that health services can draw upon to inform their health literacy champion roles, programs, and training. Champion research in the broader healthcare literature faces similar issues. For example, most studies only report on the presence or absence of a champion, and do not separate the unique effects of champions from broader multi-component implementation efforts (Miech *et al.*, 2018; Lennox *et al.*, 2020; Shea, 2021). To build a stronger evidence base, health literacy champion research must include experimental study designs and process evaluations that focus specifically on the champions themselves. This must also be accompanied by more detailed reporting (e.g. staff involved, training and expected roles). Over time we may then develop a better understanding of why a given health literacy champion initiative may or may not have worked (Powell *et al.*, 2019).

This review did identify some promising directions for health services looking to establish health literacy champions. Notably, several articles described having *multiple champions* working simultaneously in a coordinated way, with some champions being at the executive or senior leadership levels. This finding is consistent with other systematic reviews of health care champions, which reported that these ‘network’ structures may be more effective than solo champions (Miech *et al.*, 2018). Interestingly, this review identified a mix of ‘top-down’ health literacy champion networks, such as the CDC model of ‘champions’, ‘allies’, and ‘workgroup members’ described by Sørensen (Sørensen, 2021); and less hierarchical approaches such as Allott and colleagues’ (2018) champions who were nested within a community of practice and alliance network that encouraged collaboration and problem-solving with other champions. The Health Literacy Hub in Western Sydney is another useful example of how a community of practice model can support champions. Over a 5-year period, the Hub has grown to more than 1300 members, providing them with health literacy information and tools, and connecting with members via seminars, mailing lists, targeted training, and partnerships or consultation projects (Muscat *et al.*, 2023). The initiative emphasizes the role of trust, co-creation, and partnership synergy in creating an effective and sustainable community of practice. Further work is needed to inform how health services can create their own sustainable networks of health literacy champions that build staff health literacy knowledge and skills, across a variety of health service settings and organizational structures.

Current organizational health literacy resources lack detailed guidance about how to identify, prepare, and support champions. For example, champions are not mentioned in the organizational health literacy responsiveness framework (Trezona *et al.*, 2017) and the ‘Ten attributes of a health-literate organization’ only briefly mentions the need to ‘cultivate health literacy champions throughout an organization’ (Brach *et al.*, 2012). The CDC provides some greater detail, advocating that a first step to improving organizational health literacy practices is to establish champions, allies and workgroup members (Centers for Disease Control and Prevention, 2022). Although a stronger evidence base is needed for concrete recommendations, these resources could guide health services to reflect on who their champions might be, the scope of their roles, expected output, and the kind of incentives, training, or support they need. Given this review highlighted that the commitment of champions may waver, the resource could also include reflection on each champion’s personal motivation for improving health literacy, and potential incentives to maintain their commitment.

The strengths of this study were that a wide range of ‘champion’ search terms were included, across multiple databases. Limitations are that only English-language articles were captured. It is also worth noting that there is also some overlap between the concepts of ‘leaders’ and ‘champions’ (Damschroder *et al.*, 2022). Studies that reported solely on leadership support for health literacy are not captured in this review. Limitations of the primary studies were that they generally had high risk of bias and champions were often not described in detail. As a result, this review cannot provide definitive conclusions about whether champions were effective, nor in which contexts. Future systematic reviews on this emerging area of research could also consider more detailed risk of bias assessment to highlight how study designs can be further improved.

Despite the potential positive impacts of health literacy champions, this review suggests that more high-quality research on health literacy champions is needed. As a first step, quality can be improved through more comprehensive reporting on health literacy champions, including who the champions are, the training they received, and the tasks they carried out. Further effectiveness-implementation research including quantitative, qualitative, and process evaluation research across multiple sites will also contribute valuable insights into this implementation strategy. Experimental research may be particularly useful for identifying strategies to support appointed champions, such as resourcing and incentives. Engaging multiple champions at varying levels within the organization, including the executive level, is a promising future direction for this area of research.

## Supplementary Material

daad074_suppl_Supplementary_Appendix

## Data Availability

Data available within the article or its supplementary materials
