# Supplementary material for: Systematic review of health literacy champions: who, what and how?
Source: Health Promot Int. 2023 Jul 20;38(4):daad074. doi: 10.1093/heapro/daad074 (PMC10357937; doi:10.1093/heapro/daad074)
Supplement: daad074_suppl_Supplementary_Appendix [file daad074_suppl_supplementary_appendix.docx]

**Search terms by database**

**MEDLINE**

1. Health Literacy/
2. ("health literacy" or "health literate").ab. or ("health literacy" or "health literate").ti.
3. ("champion*" or "change agent*" or "opinion leader*" or "liaison*" or "liason*" or "ambassador*" or "implementation leader*" or "emergent leader*" or "promoter*" or "advocate*").ab. or ("champion*" or "change agent*" or "opinion leader*" or "liaison*" or "liason*" or "ambassador*" or "implementation leader*" or "emergent leader*" or "promoter*" or "advocate*").ti.
4. 1 or 2
5. 3 and 4
6. Limit 5 to English language.

**Embase**

1. Health Literacy/
2. ("health literacy" or "health literate").ab. or ("health literacy" or "health literate").ti.
3. ("champion*" or "change agent*" or "opinion leader*" or "liaison*" or "liason*" or "ambassador*" or "implementation leader*" or "emergent leader*" or "promoter*" or "advocate*").ab. or ("champion*" or "change agent*" or "opinion leader*" or "liaison*" or "liason*" or "ambassador*" or "implementation leader*" or "emergent leader*" or "promoter*" or "advocate*").ti.
4. 1 or 2
5. 3 and 4
6. Limit 5 to English language.

**CINAHL**

1. (MH “Health Literacy”)
2. TI ("health literacy" or "health literate") OR AB("health literacy" or "health literate").
3. TI ("champion*" or "change agent*" or "opinion leader*" or "liaison*" or "liason*" or "ambassador*" or "implementation leader*" or "emergent leader*" or "promoter*" or "advocate*") OR AB ("champion*" or "change agent*" or "opinion leader*" or "liaison*" or "liason*" or "ambassador*" or "implementation leader*" or "emergent leader*" or "promoter*" or "advocate*").
4. S1 OR S2
5. S3 and S4
6. Narrow S5 by language: English.

**Scopus**

(((ABS("health literacy" or "health literate")) OR (TITLE("health literacy" or "health literate"))) OR (KEY(“health literacy”))) AND ((ABS("champion*" or "change agent*" or "opinion leader*" or "liaison*" or "liason*" or "ambassador*" or "implementation leader*" or "emergent leader*" or "promoter*" or "advocate*") OR TITLE("champion*" or "change agent*" or "opinion leader*" or "liaison*" or "liason*" or "ambassador*" or "implementation leader*" or "emergent leader*" or "promoter*" or "advocate*"))) AND (LIMIT-TO (Language, “English”))

**PubMed**

(((“health literacy”[Title/abstract]) OR (“health literate”[Title/Abstract]) OR (health literacy[MeSH Terms])) ) AND ("champion*"”[Title/abstract] or "change agent*"”[Title/abstract] or "opinion leader*"”[Title/abstract] or "liaison*"”[Title/abstract] or "liason*"”[Title/abstract] or "ambassador*"”[Title/abstract] or "implementation leader*"”[Title/abstract] or "emergent leader*"”[Title/abstract] or "promoter*"”[Title/abstract] or "advocate*"”[Title/abstract]) Filters: English

**Appendix Table 1. Summary of risk of bias assessments, qualitative studies**

| **JBI critical appraisal criteria** | **Adsul (2017)** | **Howe (2020)** | **Kaper (2019)** | **Mabachi (2019)** |
| --- | --- | --- | --- | --- |
| Is there congruity between the stated philosophical perspective and the research methodology? | Unclear | Unclear | Unclear | Yes |
| Is there congruity between the research methodology and the research question or objectives? | Unclear | Yes | Unclear | Yes |
| Is there congruity between the research methodology and the methods used to collect data? | Unclear | Yes | Yes | Yes |
| Is there congruity between the research methodology and the representation and analysis of data? | Yes | Yes | Yes | Yes |
| Is there congruity between the research methodology and the interpretation of results? | Yes | Yes | Yes | Yes |
| Is there a statement locating the researcher culturally or theoretically? | No | No | No | No |
| Is the influence of the researcher on the research, and vice- versa, addressed? | No | No | No | No |
| Are participants, and their voices, adequately represented? | Unclear | Yes | Yes | Yes |
| Is the research ethical according to current criteria or, for recent studies, and is there evidence of ethical approval by an appropriate body? | Unclear | Yes | Yes | Yes |
| Do the conclusions drawn in the research report flow from the analysis, or interpretation, of the data? | Yes | Yes | Yes | Yes |
| **Risk of Bias** | **Moderate** | **Low** | **Low** | **Low** |

**Appendix Table 2. Summary of risk of bias assessments, quasi-experimental studies**

| **JBI critical appraisal criteria** | **Morrison (2021)** | **O'Neal (2013)** |
| --- | --- | --- |
| Is it clear in the study what is the ‘cause’ and what is the ‘effect’ (i.e. there is no confusion about which variable comes first)? | Yes | Yes |
| Were the participants included in any comparisons similar? | Unclear | Unclear |
| Were the participants included in any comparisons receiving similar treatment/care, other than the exposure or intervention of interest? | Yes | Unclear |
| Was there a control group? | No | Yes |
| Were there multiple measurements of the outcome both pre and post the intervention/exposure? | Yes | No |
| Was follow up complete and if not, were differences between groups in terms of their follow up adequately described and analyzed? | Yes | No |
| Were the outcomes of participants included in any comparisons measured in the same way? | Yes | Yes |
| Were outcomes measured in a reliable way? | Yes | Unclear |
| Was appropriate statistical analysis used? | Yes | Yes |
| **Risk of Bias** | **Low** | **High** |
